# The Many Hats of Sonic Hedgehog Signaling in Nervous System Development and Disease

**DOI:** 10.3390/jdb4040035

**Published:** 2016-12-10

**Authors:** Yesser H. Belgacem, Andrew M. Hamilton, Sangwoo Shim, Kira A. Spencer, Laura N. Borodinsky

**Affiliations:** Department of Physiology & Membrane Biology and Institute for Pediatric Regenerative Medicine, Shriners Hospital for Children Northern California, University of California Davis School of Medicine, Sacramento, CA 95817, USA; andrewmichaelhamilton@gmail.com (A.M.H.); sshim4@gmail.com (S.S.); kaspencer@ucdavis.edu (K.A.S.)

**Keywords:** non-canonical Sonic hedgehog signaling, neuronal differentiation, calcium signaling, synapse formation, neurodevelopmental disorders, medulloblastoma, neural regeneration

## Abstract

Sonic hedgehog (Shh) signaling occurs concurrently with the many processes that constitute nervous system development. Although Shh is mostly known for its proliferative and morphogenic action through its effects on neural stem cells and progenitors, it also contributes to neuronal differentiation, axonal pathfinding and synapse formation and function. To participate in these diverse events, Shh signaling manifests differently depending on the maturational state of the responsive cell, on the other signaling pathways regulating neural cell function and the environmental cues that surround target cells. Shh signaling is particularly dynamic in the nervous system, ranging from canonical transcription-dependent, to non-canonical and localized to axonal growth cones. Here, we review the variety of Shh functions in the developing nervous system and their consequences for neurodevelopmental diseases and neural regeneration, with particular emphasis on the signaling mechanisms underlying Shh action.

## 1. Introduction

The highly complex nature of the central nervous system is not only due to the copious number of cells it is composed of, but also to the number and diversity of connections between these cells. Moreover, neural function relies on dynamic changes in synapse connectivity and strength. This dynamic complexity of the nervous system is present throughout development and into adulthood. Nevertheless, different developmental stages are characterized by sets of specific cellular events, and the temporal and spatial boundaries of the developmental processes that create the nervous system are critically important. These boundaries are established by tight regulation of neural cell proliferation, neural progenitor specification, neuronal differentiation, synapse formation and neural plasticity. Intrinsic and extrinsic factors regulate these events. One potentially simple mechanism of switching from one cellular process to the next would be to switch factors by upregulating the expression of a “new” one while downregulating the expression of the “old” one. However, this paradigm does not always seem to apply; Sonic hedgehog (Shh) is a prominent example of a protein that is expressed in both the incipient nervous system and in the adult brain, but whose role varies widely throughout development. Its function in the nervous system was once thought to be restricted to the promotion of neural stem cell proliferation and neural progenitor specification. More recent studies have challenged this idea by showing that Shh signaling targets many different types of neural cells and by providing evidence of the diversity of processes regulated by Shh. Indeed, Shh appears to serve diverse functions depending on the developmental stage and maturity of the neural cells in question.

Here, we review the many roles of Shh during nervous system development and the signaling mechanisms associated with them. We propose a model of Shh signal switching throughout development to accommodate the wide variety of tasks and adapt to the dynamic changes in neural cell function as the nervous system develops and matures.

## 2. Neural Cell Proliferation

**Figure 1:**
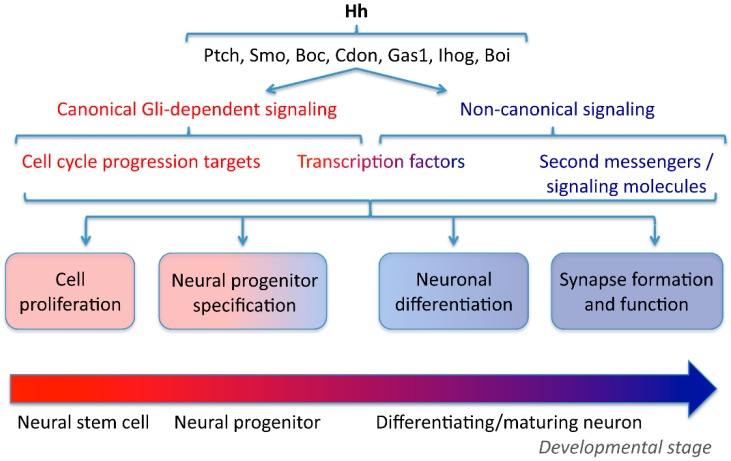
Hedgehog (Hh) signaling spans nervous system development. Hh interacts with different receptors, co-receptors and interacting proteins to recruit canonical, glioma-associated oncogene (Gli)-dependent or non-canonical signaling pathways regulating the cellular events that encompass neural development and function. Boc: brother of cell adhesion molecule-related/down-regulated by oncogenes (Cdon); Boi: brother of interference hedgehog (Ihog); Gas1: growth-arrest specific gene-1; Ptch: Patched; Smo: Smoothened.

Nervous system morphogenesis relies on the expansion of stem cells to provide the critical cell mass necessary for neural function. Production of neural cells is achieved by active proliferation of neural stem cells during development, an event promoted by canonical Shh signaling (Figure 1 and Table 1). This pathway is initiated when Hedgehog (Hh) binding to the receptor Patched (Ptch) [1,2] releases inhibition on the co-receptor Smoothened (Smo) [3,4,5], which leads to upregulation of the activity of the transcriptional activators glioma-associated oncogene (Gli) factors [6,7]. Ectopic expression of *Shh* in the mouse embryonic spinal cord approximately doubles cell proliferation in the dorsal neural tube [8], while an increase in *Gli1* levels in *Nestin*-expressing mouse neural progenitors increases precursor cell numbers in vivo, resulting in a larger brain and broader progenitor domains [9]. Shh/Gli proliferative activity is also apparent in the early postnatal mouse cerebellum, where Shh secreted by Purkinje cells promotes cerebellar granule cell precursor proliferation and prevents their differentiation [10]. In addition to Ptch, Shh proliferative action requires the Hh-binding protein Gas1 (growth arrest specific 1) and the co-receptor Boc (biregional cell-adhesion-molecule-related/downregulated by oncogenes (Cdon)-binding protein) in cerebellar granule neuron progenitors from three-day-old mice [11]. In certain structures of the postnatal and adult brain, neural stem cells continue to produce neurons. Proliferation of adult neural stem cells in mouse hippocampus depends on Shh [12], and Shh also inhibits programmed cell death, contributing to the maintenance of adult neural stem cell niches in the subventricular zone, the dentate gyrus and olfactory bulb [13]. Shh acting through Smo at the primary cilium is also necessary for the recruitment of dentate gyrus postnatal progenitors [14]. However, ablation of primary cilia during embryonic development does not disrupt neurogenesis in the ventricular-subventricular zone of neonatal mice, with the exception of the anterior ventral neural cell population [15], suggesting very specific zones of influence for Shh-regulated neuroproliferation. In the adult brain, neural stem cell proliferation and neurogenesis also appear to depend more on restricting the repressor activity of Gli3R than on the activator character of Gli transcription factors according to double-mutant experiments for *Gli2* and *Gli3* [16]. 

The mechanisms of Shh/Gli-driven promotion of neural cell proliferation involve the regulation of cell cycle kinetics and cell survival. Shh canonical signaling regulates the length of G1 phase and expression of the anti-apoptotic factor *Bcl2* in the ventral neural tube of chick embryos [17]. Similarly, reciprocal inhibitory interaction between Gli1 and p53 in neural progenitors controls neural stem cell number by enhancing proliferation and restricting stem cell expansion, respectively [9]. Shh promotes neuroepithelial cell proliferation in the embryonic chick and mouse neural tube by controlling progression into the G2 phase through regulating the expression of cyclins E, A and B [18] (Table 1). Additionally, Gli activity is necessary for Wnt-dependent control of G1 phase length in neural progenitors through upregulation of cyclin D1 expression by tcf3/4 [18].

However, regulation of neural cell proliferation by Shh is not always positive. For instance, in the developing *Xenopus* retina, Hh signaling speeds up the cell cycle by reducing the length of G1 and G2 phases. This may contribute to regulating the conversion from slow-cycling stem cells to fast-cycling transient amplifying progenitors [19]. Similarly, in *Drosophila* postembryonic brain neuroblasts, high levels of Hh signaling cause premature neuroblast cell cycle exit and underproliferation, which has consequences on the timing of neurogenesis [20].

The differences in Shh action on neural cell proliferation prompt the notion that the signaling pathways recruited by Shh are diverse and that the cellular outcome varies depending on the target cell and its maturational status, as well as on the molecular partners available at a given space and time. Indeed, Shh mitogenic and patterning functions are uncoupled when proteoglycan binding of Shh is perturbed. While this interaction is necessary for the proliferation of neural stem cells, it is not required for neural tube ventral patterning [21], providing an example of the versatility of Shh action depending on interactions with additional signaling molecules.

## 3. Neural Progenitor Specification

The transition from proliferation to differentiation in the developing nervous system occurs in a spatiotemporally-regulated manner (Figure 1). Whether Shh signaling participates in this transition remains unclear; however, profuse evidence has demonstrated that Shh function spans beyond its mitogenic role. The competence of the cell and its maturational status restrict the proliferative activity of Shh signaling, as suggested by the aforementioned study in which ectopic Shh expression in early fetal stages elicited enhanced neural cell proliferation, but not when Shh overexpression was implemented in the late stages of embryonic mouse nervous system formation [8]. Nevertheless, overexpression of Shh in late fetal stages, although not mitogenic, still halts the neural precursors in an undifferentiated state [8], indicating that Shh signaling may regulate the transitions between different neural cell maturational states.

One of the first functions attributed to the Shh pathway was its patterning role during tissue morphogenesis. In particular, Hh canonical signaling is responsible for specifying neural progenitors that in turn will give rise to different neuronal phenotypes in the developing nervous system. The patterning of ventral identities in the embryonic spinal cord has become a paradigm of the precise regulation exerted by the Shh canonical pathway [22,23,24,25,26,27]. Early studies demonstrated that blocking Shh signaling impairs the generation of motor neurons through its action during two critical periods; one in which neural plate cells are specified into ventralized progenitors and a later period during which Shh drives the differentiation of ventralized progenitors into motor neurons or interneurons [25]. The decision to adopt a certain neuronal identity is driven by a spatiotemporal code of transcription factor expression triggered by Shh levels and sensed by neural progenitors depending on their position relative to the ventrally-secreted morphogen. For instance, expression of *Nkx2.2* in ventral neural progenitors is necessary for the neurogenesis of V3 ventral interneurons in the embryonic mouse spinal cord, while its expression limits the ventral boundaries for the generation of the motor neuron domain [24], which in turn depends on the ventral neural progenitor expression of Pax6 [26] (Table 1). However, neural progenitors are not static, as they migrate along the main axes of the neural tube, suggesting that they may sense different levels of Shh throughout development. In zebrafish embryos, this results in heterogeneous populations of specified progenitors that are afterwards sorted into sharply-bordered domains, including that for motor neuron progenitors [28]. Expression of specific cell adhesion molecules facilitates this cell sorting independently of canonical Shh signaling [28]. These findings agree with the discovery that translation of the Shh gradient into patterns of transcription factor expression for specifying neural progenitors occurs through the integration of Shh levels and signaling duration [29]. The interpretation of Shh gradient dynamics relies on a transcriptional network that can store the commands coming from integrated Shh signaling to define the specialization of ventral spinal cord progenitors [30]. Expression of many of these transcription factors is regulated by Gli activity directly [31,32,33,34,35], accounting for the central role of the canonical Shh pathway in the specification of neural progenitors. An interesting aspect of the activity of these transcription factors is that they are transcriptional repressors and as such are believed to repress the expression of alternative phenotypes, thus favoring the acquisition of a specific identity [31].

Glial progenitor cell specification is also dependent on Shh. The most prominent example is the oligodendrocyte lineage, for which the embryonic specification of their precursors depends on Shh derived from the floor plate and the notochord [36,37]. The basic helix-loop-helix transcription factors that are targeted in Shh-driven specification of oligodendrocyte progenitors are Olig1 and Olig2 in the mouse embryonic spinal cord [38]. Intriguingly, Olig2 is required for both motor neuron and oligodendrocyte progenitor specification, the former being specified earlier in the embryonic spinal cord than the latter [39]. A phosphorylation in Olig2 presumably mediated by cyclic adenosine monophosphate (cAMP)-dependent protein kinase A (PKA) participates in the switch from motor neuron to oligodendrocyte precursor [40]. 

In addition to the spinal cord, the developing brain is also patterned by Shh signaling. Shh is necessary and sufficient to define the size and shape of the midbrain arcs characterized by the expression of different combinations of the transcription factors Phox2A, Gata2, FoxA2 and the stripes expressing Pax6 or Evx1 in chick embryos [41] (Table 1). High rostroventral and low caudodorsal Shh specifies thalamic progenitor cells [42,43,44,45]. Interestingly, in the medial ganglionic eminence of mouse embryos, Shh signaling participates in a feed-forward mechanism triggered by the transcription factors Lhx6 and Lhx8, upregulating the expression of Shh, which in turn drives progenitors towards the phenotype of pallial interneurons and components of the septum and bed nucleus stria terminalis [46]. During corticogenesis, Shh canonical signaling regulates the specification of different subtypes of neural progenitors that will eventually provide the differentiated neurons to populate the cortical layers [47]. However, the level of Shh signaling is tightly regulated, and the predominant Gli form is the repressor Gli3R, the primary Gli antagonist of canonical Shh signaling [48,49,50,51]. This clamping of Shh canonical signaling at low levels is crucial and appears to be dependent on the negative regulator Suppressor of Fused (SuFu), since neural progenitor cells null for SuFu fail to maintain neural progenitor identity, and this conditional SuFu knockout mouse exhibits impaired corticogenesis [52].

## 4. Neuronal and Glial Differentiation

Pioneering studies on the invertebrate model organism *Drosophila* took the lead at demonstrating the role of Hh signaling in neuronal differentiation beyond its action in cell proliferation and progenitor specialization (Figure 1). Hh secreted by differentiated neurons triggers the differentiation of the cells next in line, propagating a wave of neuronal differentiation in the developing retina [53,54,55]. This mechanism is conserved in vertebrates like zebrafish, in which Shh derived from the first retinal neurons elicits a wave of neuronal differentiation extending across the vertebrate retina [56]. 

In the developing spinal cord, Shh-driven expression of the homeobox gene MNR2 is sufficient to initiate the motor neuron differentiation program that leads to the cholinergic phenotype and characteristic axonal routing [57]. Although the specification of the motor neuron phenotype starts in the motor neuron progenitor, some aspects of motor neuron differentiation continue in the post-mitotic neuron, such as acquiring motor neuron-specific axonal trajectory [58,59], which is activity dependent [59]. Ventral neurons in the forebrain are also responsive to Shh signaling through the expression of the transcription factor Islet-1 [60].

**Figure 2:**
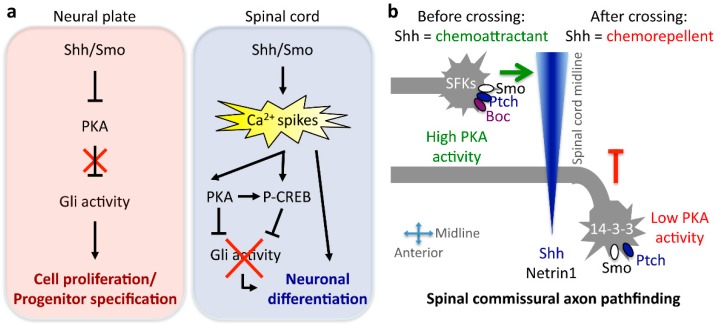
Mechanisms of Sonic hedgehog (Shh) signaling conversion during neural development. (**a**) Canonical Gli signaling is recruited in the developing neural plate, leading to neural stem cell proliferation and neural progenitor specification. The transition from neural plate to spinal cord in *Xenopus laevis* embryos is accompanied by a switch in Shh signaling from Gli to Ca^2+^ spike activity dependent, which results in the recruitment of protein kinase A (PKA) and the shutting off of Gli transcriptional activity in the differentiating neuron. (**b**) Shh action changes when commissural spinal axons cross ventrally the midline from chemoattractant to chemorepellent due to differential, growth cone-localized signaling in mouse embryos. The mediolateral and posteroanterior Shh gradient is represented in blue. P-CREB: phosphorylated cyclic adenosin monophosphate response element-binding protein.

More recent studies have demonstrated roles for Shh in the aspects of neuronal identity that develop further down the path of progenitor specification, in the postmitotic neuron. Altering Shh signaling modifies the neural activity present in developing spinal neurons of *Xenopus laevis* embryos [61] (Figure 2a and Table 1). This activity manifests as Ca^2+^ spikes that last for 10 h during development concurrent with neuronal specialization [61,62,63,64]. Intriguingly, the frequency of Ca^2+^ spike activity is higher in ventral spinal neurons compared to their dorsal counterparts [62], resembling the ventrodorsal gradient for Shh in the embryonic spinal cord and opposing the dorsoventral gradient of bone morphogenetic protein (BMP), another morphogen acting inversely to Shh. Our studies reveal that Shh and BMPs regulate Ca^2+^ spike activity in an opposing manner; Shh increases Ca^2+^ spike frequency in spinal neurons, while BMPs decrease it [61,64]. In turn, electrical activity levels regulate the expression of neurotransmitter phenotype [62,65,66,67,68]. By recruiting Ca^2+^ spike activity as part of non-canonical signaling, Shh modifies neurotransmitter specification in developing spinal neurons [61]. Enhancing Shh signaling results in greater numbers of gamma-aminobutyric acid-ergic (GABAergic) spinal neurons, while blocking Shh signaling leads to fewer GABAergic neurons. These effects are prevented by suppressing Shh signaling-induced changes in Ca^2+^ spike activity, suggesting that Ca^2+^ signaling acts downstream of Shh action on neurotransmitter specification in developing neurons [61]. These findings suggest that the interaction between Shh and Ca^2+^-dependent electrical activity adds plasticity to the process of spinal neuron differentiation.

Shh action is also relevant for glial differentiation as shown in a recent study in the developing and adult cerebellar cortex of the mouse. There, Shh produced by the neuronal populations of Purkinje and cerebellar granule cells determines the acquisition of Bergmann glial cell or velate astrocyte identities, through the regulation of the expression of specific glutamate transporters and receptors and the potassium channel Kir4.1 [69]. This study further challenges the concept that both neurons and glial cell differentiation are fully determined by a hardwired developmental program. Instead, this demonstrates that neurons, through Shh signaling, regulate their own cellular environment by dynamically changing the physiological properties of glial cells [69].

## 5. Axon Guidance

Another important functional feature of differentiating neurons is their axonal trajectory, which enables the establishment of appropriate connections with target cells. In zebrafish, Shh signaling regulates the expression of Netrin1, a known axon guidance molecule expressed by the floor plate and anterior ventral neural tube [70]. Shh effect on axon guidance is not limited to transcriptional regulation of expression of guidance cues; rather, Shh itself functions as a guidance cue by acting as a chemoattractant for commissural spinal axons in their trajectory towards the floor plate before midline crossing [71]. Attraction of commissural axons by Shh is transcription independent and Smo and Boc dependent, and leads to the activation of Src family kinases through a non-canonical signaling pathway [72,73]. Interestingly, this signaling is recruited at the growth cone of the commissural axon in a synergistic manner when both guidance cues, Netrin1 and Shh, are present, serving as a signaling mechanism for the integration of multiple cues [74]. Spinal commissural axons need to travel rostrally after crossing the midline, and this trajectory is also regulated by Shh, although instead of acting as a chemoattractant, here it acts as a chemorepellent for post-commissural spinal axons [75,76]. In chick embryos, the repulsive postcommissural axon guidance is driven through a Ptch- and Smo-independent mechanism mediated by hedgehog interacting protein (Hhip) [75]. In contrast, in the mouse embryonic spinal cord, genetic experiments showed that Hhip is dispensable for Shh-mediated repulsion, while Smo is required for Shh chemorepulsive action [76]. Moreover, the switch in commissural axon responsiveness to Shh is dependent on the developmentally-regulated expression of 14-3-3 proteins that regulate the activity of binding partners, such as PKA, resulting in decreased PKA activity in post-crossing axonal growth cones [76] (Figure 2b).

The Shh effects on axon guidance are also apparent in retinal ganglion cells. First, it was shown that axonal growth was negatively affected by Shh when growing towards the diencephalic ventral midline in the chick embryo [77]. Further studies showed that in vitro exposure to low and high levels of Shh attracts or repels retinal ganglion cell axons from chick embryos, and in vivo overexpression or inhibition of Shh both result in loss of centrally-directed retinal ganglion cell axon projections towards the optic disc [78]. In mice, Shh repels retinal ganglion cell axons, an action mediated by Boc and necessary for the normal segregation of these projections at the optic chiasm [79]. Shh-driven axon guidance can also be indirect, through regulating the expression of factors directly involved in axon guidance; these factors include Slit guidance molecules, which regulate the zebrafish forebrain midline crossing of postoptic commissural axons [80], and the chemokine pathway protein stromal cell-derived factor 1, which is expressed in the optic stalk in a Shh-dependent manner and is necessary for regulating the guidance of retinal ganglion cell axons [81].

In addition to central nervous system axons, Shh regulates the axonal trajectory of enteric neurons that innervate the smooth muscle in the gastrointestinal system. Here, Shh action consists of repelling enteric axons from projecting towards the central villi, allowing for tubular innervation of the gut wall in mouse embryos [82]. In this case, the signaling downstream of Shh includes the binding partner Gas1 and Smo expressed in enteric neurons, which in turn recruit the G protein α Z [82]. 

All of these studies demonstrate that Shh signaling through noncanonical mechanisms targets postmitotic differentiating neurons to regulate a number of traits, including neurotransmitter phenotype specialization, axonal morphology and axon guidance. 

## 6. Synapse Formation and Plasticity

Once axons reach their targets, they establish synapses, which constitute the circuitry that enables neural network function (Figure 1). During *Drosophila* visual system development, Hh is delivered from photoreceptor axons to the lamina ganglion layer, inducing differentiation of their synaptic partners, the lamina neurons. Hedgehog is localized to axons, growth cones and synapses by a conserved motif in the hedgehog C-terminus [83]. The establishment of the retinotopic map through the formation of specific synapses relies on Hh-induced expression of the transcription factor Single-minded in lamina neurons, which enables association between the pre- and post-synapse by as yet unclear mechanisms [84]. Another subset of synapses in which partners are matched through Hh signaling intervention are the connections between olfactory receptor neurons and their glomerular targets in the *Drosophila* brain. First, Hh leads to differential Ptch expression in peripheral sensory organ precursors, which leads to olfactory neurons with different responsiveness to hedgehog derived from the brain [85]. Hence, in a second step of Hh intervention, only olfactory neurons expressing low levels of Ptch will depend on Hh derived from brain neurons for their axonal targeting through recruitment of Smo and the coreceptor Interference hedgehog (Ihog), a mechanism not available in high Ptch-expressing olfactory neurons due to strong inhibition of Smo [85].

Hh signaling components present in axonal terminals and postsynaptic specializations seem to be an aspect conserved across species. In addition to *Drosophila*, Smo is found presynaptically in close proximity to synaptic vesicles in the hippocampal mossy fibers of adult mice [86], and in the rat hippocampus, Ptch and Smo localize to processes and growth cones in developing neurons and in dendrites, dendritic spines and postsynaptic sites of the mature hippocampus [87]. Shh is present in inhibitory and excitatory synapses in the region I and III of hippocampus proper (CA1 and CA3) and dentate gyrus of the adult rat hippocampus, in pre- and post-synaptic neurons in the vicinity of the active zone, but in distinctly extrasynaptic sites [88]. Shh is also expressed in layer V corticofugal projection neurons, and its coreceptor Boc is expressed in local and callosal projection neurons of layer II/III, whose axons synapse onto subcortical projection neurons [89]. Moreover, the number of synapses formed onto layer V neurons and the strength of these synapses depend on expression of Shh and Boc, respectively [89], indicating the relevance of Shh signaling for synapse formation and function in vertebrates. In agreement with these findings, presynaptic terminals of cultured hippocampal neurons increase in size upon Shh exposure, resulting in increased frequency of miniature postsynaptic currents [90]. 

## 7. Interactions of Shh with Other Signaling Pathways in the Nervous System

**Figure 3:**
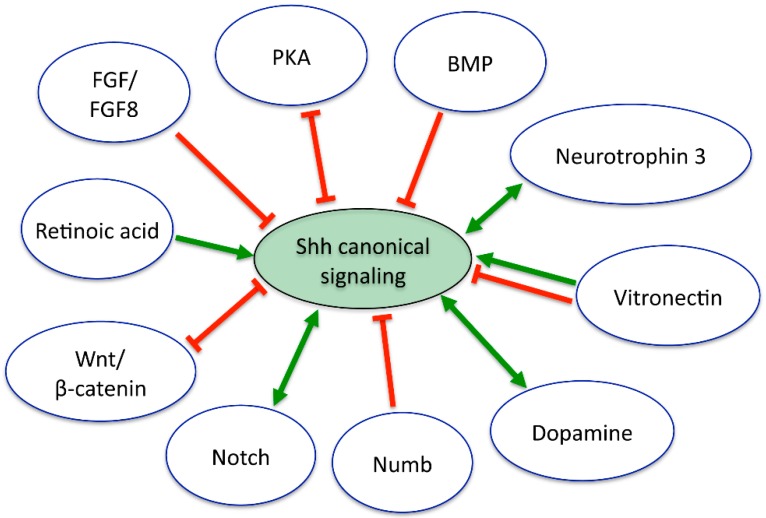
Multiple interactions between Shh and concurrent developmental cues modulate the Shh canonical signaling. Red and green arrows represent antagonistic and synergistic interactions, respectively. Double arrows represent feedback loops. See the text for details.

Shh as a developmental factor for the nervous system is not spatiotemporally isolated, but rather acts concurrently with numerous cues and signaling pathways that dynamically change as the neural tissue develops and matures. Hence, Shh signaling is subjected to modulation by other developmentally-regulated pathways, which may even change Shh signaling’s own action in the developing nervous system. Indeed, for Shh to participate seamlessly in the diverse, and sometimes contrasting, cellular events referred to in the above sections as nervous system development progresses, Shh activity needs to change with the maturational status of the neural cell. Here, we review some of the most prominent interactions of Shh with other signaling pathways and the modifications that result in the mechanisms of action for Shh signaling itself during nervous system development (Figure 3). 

Rostrally to vertebrate mid-diencephalon, BMP7 and Shh are expressed from the same prechordal mesoderm and act on neural cells coordinately to induce the differentiation of rostral diencephalic ventral midline cells. BMP7 modifies Shh action by shifting the specification towards rostral phenotypes that otherwise, in the absence of BMP7, differentiate into Shh-producing floor plate cells [91]. In chick neural plate explants, BMP signaling modifies the responses of neural cells to Shh by inducing a ventral-to-dorsal switch, antagonistic activity that is modulated in vivo by the expression of BMP inhibitors along with Shh in structures ventral to the neural tube, like the notochord [92]. Another example of signaling interaction demonstrated through in vitro and ex vivo studies from mouse embryonic spinal cord is the synergy between Shh and neurotrophin 3 on motor neuron differentiation, through the regulation of Islet1 expression [93]. Accordingly, neurotrophin 3 knockout mice lack the subclass of fusimotor neurons, which make up 30%–40% of total spinal motor neurons [94]. These findings suggest that the differentiation of some subclasses of motor neurons requires interaction between Shh and neurotrophin 3 [93].

Since most of the transcription factors regulated by Shh are repressors of gene transcription, it is essential to consider that additional signaling pathways must be needed for the induction of a specific phenotype. Indeed, retinoic acid signaling is required for Shh-dependent motor neuron differentiation, while FGF8 downregulates the expression of Shh-induced repressors [95]. Another synergistic partner of Shh is the extracellular matrix protein Vitronectin, which is coexpressed with Shh in the chick notochord, floor plate and ventral neural tube and enhances the differentiation into motor neurons of cultured neuroepithelial cells only when coincubated with Shh, presumably by optimizing Shh presentation to target cells [96]. In contrast, in cerebellar granule cells grown in vitro, Vitronectin overrides the Shh proliferative signal to induce neuronal differentiation by recruiting the transcription factor cyclic-AMP responsive element-binding protein (CREB) [97]. Similarly, FGF promotes differentiation of cerebellar granule neurons in vivo and in vitro by recruiting ERK and JNK kinases and preventing Gli activity, thus abolishing Shh-induced proliferation. Moreover, FGF stops proliferation of *patched* mutant mice-derived tumor cells [98]. In some cases, the interaction between Shh and other signaling factors follows parallel pathways until they intersect at the level of transcriptional regulation, as illustrated by the establishment of thalamic neuron identity defined by a balance of transcription factor expression; thalamic Wnt/β-catenin signaling results in the expression of Neurogenin1/2, while its repressor Nkx2.2 is driven by Shh–Gli signaling, defining the domains of thalamic neuron progenitors through mutual regulation of the transcriptional network [99].

Canonical Gli-dependent Shh signaling is also negatively regulated by PKA, and in the mouse neural tube, this regulation is implemented at the basal body of the primary cilium [100]; thus, pathways that inhibit PKA activity may enhance Gli activity. One interesting example of this interaction is the dopaminergic innervation from the brainstem that promotes motor neuron specification at the expense of V2 interneurons in the embryonic zebrafish spinal cord. This interaction between dopaminergic and Shh canonical signaling happens through activation of D4a dopamine receptors in pre-motor neuron progenitors that inhibit adenylate cyclase, lowering cAMP levels and inhibiting PKA. This results in Gli2 upregulation and increases the number of spinal motor neurons in the developing zebrafish [101]. A similar mechanism is activated upon injury when upregulated dopamine signaling promotes motor neuron regeneration by enhancing Shh signaling [101].

Another interesting type of interaction of signaling pathways with Shh emerges from regulating the localization of Shh signaling components (Table 1). Notch signaling inhibits Ptch localization to the primary cilium, allowing for Smo translocation to this subcellular compartment for full activation of Gli transcription factors, synergizing with Shh activity in regulating neural tube cell specification [102]. In contrast, Numb exerts an antagonistic role on Gli transcriptional activity by binding and targeting Gli to the proteasome-dependent degradation pathway through E3-ubiquitin ligase Itch during cerebellar granule cell progenitor differentiation [103]. Additionally, Notch is known to maintain neural stem cell character and prevent neuronal differentiation and is also inhibited by Numb through ubiquitin-dependent processing of Notch1 [104]. Thus, the obligatory role of Numb in neurogenesis seems to rely on inhibiting Shh proliferative action and Notch signaling [102,105]. Multiple functions of Shh in nervous system proliferation, differentiation and patterning may be facilitated by coordinated regulation and crosstalk between other signaling pathways that include Notch, Numb, BMP and Wnt (Figure 3).

The examples of interacting signaling pathways reviewed thus far result in promoting or preventing the canonical Gli-driven Shh pathway. However, considering that Shh expression persists throughout development and adulthood exerting diverse functions, there is ample support for the model of Shh signaling switching from one pathway that promotes proliferation to others that enable differentiation, maturation and survival of neural cells. In addition to dynamic changes in canonical Shh signaling, Shh becomes engaged in other non-canonical signaling cascades as development progresses, a concept that we recently demonstrated in the embryonic *Xenopus* spinal cord. Gli expression and activity decrease abruptly during spinal cord development when transitioning from the neural plate to spinal cord in *Xenopus* embryos [106,107] and in mice, approximately 30 h post-headfold [30] in nine-day-old embryos [106]. This downregulation of Gli activity is concurrent with the appearance of Ca^2+^ spikes in embryonic *Xenopus* spinal neurons, the frequency of which is enhanced by Shh in a transcription-independent, phospholipase C- and transient receptor potential channel-dependent manner [61]. In turn, Ca^2+^ spikes enhance PKA activity, thus switching Shh action from inhibiting PKA activity in the neural plate to enhancing it in differentiating spinal neurons [107]. This results in a paradoxical turning off of Shh’s own canonical Gli-driven signaling during spinal cord development and a switch in Shh signaling to a non-canonical electrical activity and PKA-mediated pathway. The mechanisms of this switch operate on several levels, through inhibiting Gli2 nuclear translocation, favoring Gli2 and Gli3 processing towards repressor forms, and by Shh-dependent activation of CREB, which in turn represses Gli1 expression [107] (Figure 2a).

## 8. Neural Disease and Regeneration

Given the many important roles that Shh plays during nervous system development, it is not surprising to find that altering Shh signaling contributes to serious neurodevelopmental diseases. Indeed, reduced canonical Shh signaling leads to holoprosencephaly [108], likely due to aberrant patterning and deficient neural cell proliferation. On the other hand, constitutive activation of this pathway by deficient Ptch function [109] underlies the occurrence of several cancers, including a subgroup of the pediatric brain tumor medulloblastoma [108,110]. An increase in Gli1 levels in neural progenitors enables the expansion of neural precursors and neural stem cells and represses expression of p53, an inhibitor of Gli1 activity [9]. The cerebellar granule neuron precursors seem to be responsible for originating the Shh-dependent medulloblastoma [111,112]. In addition to N-myc, a common proto-oncogene [113], another transcription factor that participates in the canonical Shh signaling-driven promotion of neuronal precursor expansion is Atoh1 [114]; Shh prevents Atoh1 degradation by the E3 ubiquitin ligase Huwe1, which results in medulloblastoma when knocked out in mice [115]. Opposing the Shh signaling-driven medulloblastoma cell proliferation and progression is the G protein Gαs, which localizes to the primary cilium in granule neuron precursors and downregulates canonical Shh signaling through a cAMP-driven pathway [116]. One of the hallmarks and critical events in the progression of medulloblastoma is the loss of Ptch1 expression, which deregulates Shh signaling [117,118,119]. The Shh binding protein Boc is upregulated in medulloblastomas and contributes to the cerebellar granule cell precursor expansion by facilitating aberrantly high Shh signaling, which leads to increased CyclinD1 activity and Ptch1 loss of heterozygosity [120].

In stark contrast to the Shh-dependent overproliferation leading to cerebellar tumors lies the phenotype afflicting Down syndrome patients and the segmentally trisomic postnatal mouse model for Down syndrome (Ts65Dn), which exhibit a smaller cerebellum due to a reduction in the number of granule neurons [121,122,123]. In Ts65Dn mice, the reduction in granule neurons results from deficient Shh-dependent mitogenic signaling in cerebellar granule neuron precursors during early postnatal development [124]. In an in vitro model, neural precursor cells from the subventricular zone and hippocampus of Ts65Dn mice show reduced canonical Shh signaling, which appears to be rooted in overexpressed Ptch1 due to transcriptional activation of Ptch1 expression by the amyloid precursor protein intracellular domain (AICD), a transcription-promoting fragment of amyloid precursor protein [125]. Moreover, treating newborn trisomic mice with the Smo agonist SAG restores cerebellar granule cell proliferation in one-week-old pups [124] and normal cerebellar morphology in adults [126]. Remarkably, SAG treatment also rescues behavioral deficits dependent on hippocampal function in the Down syndrome mouse model and partially restores theta burst stimulation-induced long-term potentiation in hippocampal slices from these animals [126]. It follows that Down syndrome, as well as other neurodevelopmental disorders, may be responsive to therapeutic intervention targeted to Shh signaling.

Regeneration of damaged neural tissue relies on many cellular events characteristic of neural development. Thus, it is not unexpected to find that Shh appears to be a vital regulator of post-injury neural regeneration. Indeed, Shh and Smo expression are upregulated in facial motor neurons after facial nerve axotomy in adult rats, and cyclopamine, a Smo antagonist, leads to a decrease in the number of surviving motor neurons after injury [127]. Similarly, after zebrafish spinal cord transection, there is an increase in Shh expression in the floor plate and upregulation of Ptch1 and Smo in ependymoradial glial cells, as well as the number of cells expressing the pre-motor neuron markers Olig2, Nkx6.1 and Pax6, which results in motor neuron regeneration, an effect that is prevented by Smo inhibitor cyclopamine [128]. In mice, invasive injury to the somatosensory cortex results in a regenerative response from astrocytes as a consequence of an inflammation-induced reactive gliosis that upregulates Shh signaling, which, in turn, leads to the generation of neural stem cells in the site of injury [129,130]. Moreover, enhancing Shh signaling by treating post-ischemic stroke mice with the Smo agonist purmorphamine is neuroprotective and improves recovery [131]. Intriguingly, recruitment of adult neural stem cells from the subventricular zone to demyelinating regions in the mouse forebrain is enhanced when the canonical Shh effector Gli1 is inhibited, while eliminating Smo-mediated signaling is detrimental for oligodendrocyte differentiation and remyelination [132]. These findings suggest that similarly to development, appropriate regeneration may require a switch from canonical to non-canonical Shh signaling. 

## 9. Conclusions

Shh is a fascinating signaling molecule that is paramount in regulating nervous system development and function. Its relevance casts it as a target of interest for therapeutics of several neurodevelopmental diseases. However, every aspect of its signaling has some intricacy that demands close investigation, from its delivery and receptor molecules, to the subcellular localization of its signaling components, including the participation of the primary cilium in orchestrating Shh signaling in vertebrates, and the downstream pathways. Future challenges include understanding how Shh signaling transitions between functions during nervous system development and identifying the mechanisms that are targeted in disease and the regeneration of neural tissue. Taking into account that there is far more to Shh signaling than its mitogenic action and neural progenitor specification will allow for further discoveries of novel mechanisms mediated by Shh during neural development, disease and regeneration. 

## Figures and Tables

**Table 1:** Mechanisms and functions of canonical and non-canonical Hedgehog (Hh) signaling in neural development.

	Canonical			Non-Canonical		
	Neural Cell Proliferation	Neural Progenitor Specification	Axon Guidance	Neuronal Differentiation	Axon Guidance	Axon Guidance
**Receptors**	Ptch, Smo (Gas1, Boc)	Ptch, Smo	Ptch, Smo	Ptch, Smo	Smo, Boc	(1) Smo (mouse), Hhip (chick) (2) Boc (3) Smo, Gas1
**Second messengers**				IP3, Ca^2+^		(1) 14-4-3, cAMP (3) Gnaz
**Transcription factors**	Gli	Gli	Gli	cJun	Transcription independent	Transcription independent
**Targets**	Bcl2, P53, cyclin A, B, E, D1	Nkx2.2, Nkx6.1, Pax6, Evx1, Phox2A, Gata2, Fox2A, etc.	(1) Slit (2) Stromal cell-derived factor 1	Tlx3	Src	
**Subcellular needs for Hh signaling**	Nucleus Primary cilium?	Nucleus Primary cilium	Nucleus	Nucleus Primary cilium?	Axonal growth cone	Axonal growth cone
**Main roles**	Regulation of cell cycle progression and cell survival	Spinal cord and brain patterning during morphogenesis	(1) Midline crossing forebrain commissural axons (2) Retinal ganglion cell axon guidance	Specification of spinal neuron transmitter phenotype	Attractant guidance commissural spinal axons	(1) Repulsive guidance commissural spinal axons (2) Repulsive retinal ganglion cell axon guidance (3) Repulsive guidance enteric axons

?: Not yet determined or neural structure dependent. Bcl2: B-cell lymphoma 2; Boc: brother of cell adhesion molecule-related/down-regulated by oncogenes; cAMP: cyclic adenosine monophosphate; Evx1: even-skipped homeobox 1; Fox2A: forkhead box protein A2; Gas1: growth arrest specific 1; Gnaz: G Protein Subunit α Z; Hhip: hedgehog interacting protein; IP3: inositol 1,4,5-trisphosphate receptor type 3; Pax6: paired box 6; Phox2A: paired like homeobox 2A; Ptch: Patched; Smo: Smoothened; Src: proto-oncogene tyrosine-protein kinase; Tlx3: T-cell leukemia homeobox 3.
